# International practices in the dietary management of fructose 1-6 biphosphatase deficiency

**DOI:** 10.1186/s13023-018-0760-3

**Published:** 2018-01-25

**Authors:** A. Pinto, M. Alfadhel, R. Akroyd, Y. Atik Altınok, S. M. Bernabei, L. Bernstein, G. Bruni, G. Caine, E. Cameron, R. Carruthers, B. Cochrane, A. Daly, F. de Boer, S. Delaunay, A. Dianin, M. Dixon, E. Drogari, S. Dubois, S. Evans, J. Gribben, G. Gugelmo, C. Heidenborg, I. Hunjan, I. L. Kok, B. Kumru, A. Liguori, D. Mayr, E. Megdad, U. Meyer, R. B. Oliveira, A. Pal, A. Pozzoli, R. Pretese, J. C. Rocha, S. Rosenbaum-Fabian, J. Serrano-Nieto, E. Sjoqvist, C. Timmer, L. White, T. van den Hurk, M. van Rijn, H. Zweers, M. Ziadlou, A. MacDonald

**Affiliations:** 1 Birmingham Women’s and Children’s Hospital, Birmingham, UK; 2 King Abdullah International Medical Research Centre, King Saud bin Abdulaziz University for Health Sciences, Genetic Division, Department of Pediatrics, King Abdulaziz Medical City, Ministry of National Guard-Health Affairs (NGHA), Riyadh, Saudi Arabia; 3 National Metabolic Service, Starship Child Health and Auckland City Hospital, Auckland, New Zealand; 4 Pediatric Metabolism Department, Ege University Medical Faculty, Izmir, Turkey; 5 Children’s Hospital Bambino Gesù, Division of Artificial Nutrition, Rome, Italy; 6 IMD Nutrition, Children’s Hospital Colorado, Aurora, CO USA; 7 Azienda Ospedaliero Universitaria Meyer, Florence, Italy; 8 Mid Yorks NHS Trust, Yorkshire, UK; 9 Bristol Royal Hospital for Children, University Hospitals Bristol NHS Foundation Trust, Bristol, UK; 10 Charles Dent Metabolic Unit, National Hospital for Neurology and Neurosurgery, London, UK; 11 Royal Hospital for Children, Glasgow, Scotland UK; 12 Division of Metabolic Diseases, Beatrix Children’s Hospital, University Medical Center Groningen, PO BOX 30.001, 9700 RB Groningen, The Netherlands; 13 Centre hospitalier Universitaire de Rennes, Rennes, France; 14 Department of Pediatrics, Regional Centre for Newborn Screening, Diagnosis and Treatment of Inherited Metabolic Diseases and Congenital Endocrine Diseases, University Hospital of Verona, Verona, Italy; 15 Great Ormond Street Hospital for Children NHS Foundation Trust, London, UK; 16 Unit of Metabolic Diseases, Choremio Research Laboratory, 1st Department of Paediatrics, University of Athens Medical School, “Aghia Sophia” Children’s Hospital, Athens, Greece; 17 Centre de référence des maladies héréditaires du métabolisme, Hôpital Necker Enfants Malades, Paris, France; 18 Evelina London Children’s Hospital, Guy’s and St Thomas’ NHS Foundation Trust, London, UK; 19 Karolinska University Hospital, Stockholm, Sweden; 20 Bradford Teaching Hospital NHS Foundation Trust, Bradford, UK; 21 Wilhelmina Children’s Hospital, University Medical Centre Utrecht, Utrecht, The Netherlands; 22 Department of Nutrition and Diet, Gaziantep Cengiz Gökçek Obstetrics and Pediatric Hospital, Gaziantep, Turkey; 23 Ernährungsmedizinische Beratung, Universitätsklinik für Kinder- und Jugendheilkunde, Salzburg, Austria; 24 Metabolic Nutrition Clinics. King Faisal Specialist Hospital and Research Centre, Riyadh, Saudi Arabia; 25 Clinic of Paediatric Kidney, Liver and Metabolic Diseases, Medical School Hannover, Hannover, Germany; 26 Centro de Referência em Erros Inatos do Metabolismo, Universidade Federal de São Paulo, São Paulo, Brazil; 27 Akademiska children’s hospital, Uppsala, Sweden; 28 Guglielmo Da Saliceto’s Hospital, Piacenza, Italy; 29 Fondazione MBBM, San Gerardo Hospital, Monza, Italy; 30 Centro de Referência de Doenças Hereditárias do Metabolismo, Centro Hospitalar do Porto – CHP, Porto, Portugal; 31 Centro de Genética Médica, Centro Hospitalar do Porto (CHP), Porto, Portugal; 32 Faculdade de Ciências da Saúde, Universidade Fernando Pessoa, Porto, Portugal; 33 Centre for Health Technology and Services Research (CINTESIS), Porto, Portugal; 34 University Children’s Hospital Freiburg, Freiburg, Germany; 35 Hospital Regional Universitario Málaga, Málaga, Spain; 36 Children’s Hospital, University Hospital, Lund, Sweden; 37 Academic Medical Center Amsterdam, Amsterdam, Netherlands; 38 Sheffield Children’s Hospital, Sheffield, UK; 39 Radboud University Medical Centre Nijmegen, Nijmegen, The Netherlands; 40 Nutrition Department at International campus, Shahid Sadoughi University of Medical Sciences, Yazd, Iran

**Keywords:** Fructose 1,6 bisphosphatase deficiency, Dietary restrictions, Fasting tolerance, Uncooked cornstarch

## Abstract

**Background:**

In fructose 1,6 bisphosphatase (FBPase) deficiency, management aims to prevent hypoglycaemia and lactic acidosis by avoiding prolonged fasting, particularly during febrile illness. Although the need for an emergency regimen to avoid metabolic decompensation is well established at times of illness, there is uncertainty about the need for other dietary management strategies such as sucrose or fructose restriction. We assessed international differences in the dietary management of FBPase deficiency.

**Methods:**

A cross-sectional questionnaire (13 questions) was emailed to all members of the Society for the Study of Inborn Errors of Metabolism (SSIEM) and a wide database of inherited metabolic disorder dietitians.

**Results:**

Thirty-six centres reported the dietary prescriptions of 126 patients with FBPase deficiency. Patients’ age at questionnaire completion was: 1-10y, 46% (*n* = 58), 11-16y, 21% (*n* = 27), and >16y, 33% (*n* = 41). Diagnostic age was: <1y, 36% (*n* = 46); 1-10y, 59% (*n* = 74); 11-16y, 3% (*n* = 4); and >16y, 2% (*n* = 2). Seventy-five per cent of centres advocated dietary restrictions. This included restriction of: high sucrose foods only (*n* = 7 centres, 19%); fruit and sugary foods (*n* = 4, 11%); fruit, vegetables and sugary foods (*n* = 13, 36%). Twenty-five per cent of centres (*n* = 9), advised no dietary restrictions when patients were well. A higher percentage of patients aged >16y rather than ≤16y were prescribed dietary restrictions: patients aged 1-10y, 67% (*n* = 39/58), 11-16y, 63% (*n* = 17/27) and >16y, 85% (*n* = 35/41). Patients classified as having a normal fasting tolerance increased with age from 30% in 1-10y, to 36% in 11-16y, and 58% in >16y, but it was unclear if fasting tolerance was biochemically proven. Twenty centres (56%) routinely prescribed uncooked cornstarch (UCCS) to limit overnight fasting in 47 patients regardless of their actual fasting tolerance (37%). All centres advocated an emergency regimen mainly based on glucose polymer for illness management.

**Conclusions:**

Although all patients were prescribed an emergency regimen for illness, use of sucrose and fructose restricted diets with UCCS supplementation varied widely. Restrictions did not relax with age. International guidelines are necessary to help direct future dietary management of FBPase deficiency.

## Background

Fructose 1, 6 bisphosphatase (FBPase) deficiency (OMIM #229700), is a rare, autosomal recessive inherited disorder of gluconeogenesis [1]. It was first described by Baker and Winegrad in 1970 [2], who found recurrent episodes of hypoglycaemia and lactic acidosis in a 5-year-old girl; her elder brother died at the age of 6 months following severe metabolic acidosis probably with the same condition [2]. FBPase is a key gluconeogenic enzyme that irreversibly catalyses the hydrolysis of fructose-1,6-biphosphate to fructose-6-phosphate and inorganic phosphate [3]. Its inactivity prevents the endogenous formation of glucose from precursors including lactate, glycerol, fructose, and gluconeogenic amino acids such as alanine [4]. Consequently alanine, lactate, glycerol, glycerol-3-phosphate and ketones [5] accumulate during fasting. Hypoglycaemia occurs after prolonged fasting when glycogen stores are depleted [3]. FBPase incidence is unknown, but is possibly between 1: 350,000 and <1: 900,000 in Europe [1, 6]. Cases have also been identified in Japan [3], China, USA [7], Israel [8], Turkey [9], Morocco, and Saudi Arabia [10]. Incidence is higher in countries with greater rates of consanguinity [11].

Clinical presentation may be as early as 1-4 days of life, when glycogen stores are limited, and is associated with a high mortality [6], but many children experience recurrent acute episodes before diagnosis is made [4]. Symptoms include hypoglycaemia, metabolic acidosis, lactic acidosis, ketonuria, apnoea, seizures, severe hyperventilation, hypotonia, and moderate hepatomegaly [12, 13], usually accompanying prolonged fasting associated with illness [14].

Dietary management is not well characterised. Management aims to prevent hypoglycemia and lactic acidosis by avoiding prolonged fasting and excessive intake of fructose and sucrose, particularly in young children. Although fructose is a gluconeogenic precursor, the severity or need for sucrose and fructose restriction remains indeterminate. It is often reported that fasting should not exceed 8 h, but it is accepted that fasting tolerance improves with age [2, 15]. It is unclear if dietary strategies such as the use of uncooked cornstarch (UCCS) to limit overnight fasting is necessary or even effective. Treatment of metabolic decompensation with oral or intravenous glucose ± sodium bicarbonate is essential. Between episodes of acute metabolic decompensation, patients usually remain well and it is considered a benign condition once diagnosis and management is established [4, 16].

Due to the rarity of the disorder, few metabolic centres manage more than 10 patients. In order to try and understand long-term dietary prescriptions in FBPase deficiency, we have identified the differences in dietary management of a large international patient cohort.

## Methods

A cross sectional questionnaire (power point document) was sent to all members of the Society for the Study of Inborn Errors of Metabolism (SSIEM) by email as well as an extensive database of IMD dietetic colleagues (*n* = 250) who have collaborated on previous surveys [17, 18].

The questionnaire was comprised of 13 open and multiple-choice questions about dietary practices in FBPase deficiency. Information on: number of patients, ethnicity, age at diagnosis, dietary restrictions, UCCS, fasting tolerance and emergency regimens was collected. Questionnaire responses were divided by geographical region in order to analyse for any effect on dietary practices. Ethical consent was not sought as outcome data and patient identifiable data was not obtained. Data was analysed using descriptive statistics only.

## Results

### Participants

Thirty-six international IMD centres from 15 countries returned questionnaires, reporting 126 patients with FBPase deficiency. The majority of responses were from dietitians (92%, *n* = 36) with a minority from physicians (8%, n = 3). Forty-five patients were Caucasian, 38 Arabic and 27 Asian (23 Pakistani and 4 Bangladeshi). The remaining 16 patients belonged to other ethnicities that were not identified. There was a median of 2 (1-24) patients per centre.

The centres were divided into the following geographical regions: Western Europe: 9 centres caring for *n* = 18 patients (Austria [*n* = 1], France [*n* = 6], Germany [*n* = 2] and The Netherlands [*n* = 9]); Southern Europe: 9 centres caring for *n* = 35 patients (Greece [*n* = 8], Italy [*n* = 13], Spain [*n* = 1] and Turkey [n = 13]); Northern Europe: 12 centres caring for *n* = 41 patients (Sweden [n = 3] and United Kingdom [*n* = 38]), and non-Europe: 6 centres caring for *n* = 32 patients (Iran [n = 3], Saudi Arabia [*n* = 25], Brazil [*n* = 2], United States of America [*n* = 1] and New Zealand [*n* = 1]).

Patient’s age at diagnosis was: < 1y, 36% (*n* = 46); 1-10y, 59% (*n* = 74), 11-16y, 3% (*n* = 4), and >16, 2% (*n* = 2). Patients were diagnosed in adulthood in Brazil and USA. Data was not collected on age of first clinical symptoms although several centres commented there was a delay between age of presentation and diagnostic age. At the time of questionnaire completion, patient age was categorised into the following groups: 46% (*n* = 58) 1-10y, 21% (*n* = 27) 11-16y; and 33% (n = 41) >16y.

### Dietary restrictions when well (Table 1)

Most patients in 75% of centres (*n* = 27/36) were prescribed sucrose or fructose restrictions; 7 centres restricted high sucrose foods only (*n* = 17 patients), 4 centres (30 patients) restricted sugary foods and fruits only and 13 centres (n = 44 patients) restricted fruits, vegetables and sugary foods. In 3 centres (9 patients), dietary prescription varied according to clinical signs and symptoms. Three centres from Western Europe, Northern Europe and Non-Europe calculated fructose amounts. More adults than children were prescribed dietary restrictions (age 1-10y, 67% [*n* = 39/58], 11-16y, 63% [*n* = 17/27] and >16y, 85% [*n* = 35/41]).

**Table 1:** Number of patients prescribed each dietary restriction by geographical region

Geographical region (Number of centres)	Patient number (n)			
	No dietary restriction	Sugary foods, fruit and vegetables restriction	Sugary foods and fruit restriction	Only sugary foods restriction
Western Europe (*n* = 18)	3	9	5	1
Southern Europe (*n* = 35)	3	19	0	13
Northern Europe (*n* = 41)	28	11	1	1
Non-Europe (*n* = 32)	1	5	24	2
Totals (*n* = 126)	35	44	30	17

Twenty-five percent of centres (*n* = 9, 35 patients) did not advocate any dietary restrictions when well. Nineteen patients were aged 1-10y, 10 aged 11-16y and 6 patients aged >16y. Dietary restrictions by geographical region are shown in Table 1. One patient was tube fed due to comorbidities.

### Fasting tolerance and use of uncooked cornstarch

**Fig. 1:**
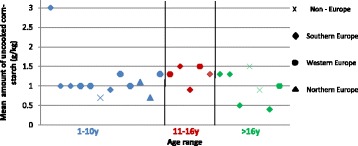
Mean amount of uncooked corn-starch (g/kg) prescribed per dose by each centre from different geographical regions in each age range

Professionals reported fasting tolerance in 112 (89%) patients, but it was unclear on what criteria this was established. Fasting tolerance improved with age, with the number of patients with reported normal fasting tolerance in each age group increasing from 30% (*n* = 14/47) in 1-10y, to 36% (*n* = 9/25) in 11-16y and 58% (*n* = 23/40) in >16y. Twenty (56%) centres (Table 2) routinely prescribed UCCS to extend overnight fasting, which represented 37% (*n* = 47/126) of all patients; but still most patients (*n* = 79) were managed without UCCS prescription. In the 47 patients prescribed UCCS, the mean dose (g/kg/dose) for the group was 1.1 and by age category was aged 1-10y (*n* = 30), 1.2; 11-16y (*n* = 7); 1.3; >16y (*n* = 10), 1.0. Figure 1 shows the mean amount of UCCS dose given in each age range in centres from different geographical regions. Two centres prescribed Glycosade (Vitaflo®), a slow release starch. Other complex carbohydrates such as cereals, bread, rice or pasta were encouraged as alternatives to UCCS before bedtime.

**Table 2:** Number of centres prescribing uncooked cornstarch by geographical region

Geographical region (Number of centres)	Number of centres (n)	
	Number of centres prescribing uncooked cornstarch	Number of centres not prescribing uncooked cornstarch
Western Europe (*n* = 9)	4	5
Southern Europe (*n* = 9)	8	1
Northern Europe (*n* = 12)	5	7
Non-Europe (*n* = 6)	3	3
Totals (*n* = 36)	20	16

**Fig. 2:**
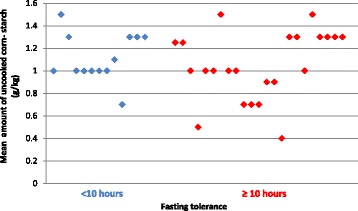
Mean dose of uncooked corn-starch (g/kg) prescribed per dose for each patient when fasting time is below and above 10 h

Sixteen centres (*n* = 53 patients) did not use UCCS before overnight fasting (7 centres from Northern Europe, 5 centres from Western Europe, 3 non-European centres [including 24 patients from one centre] and 1 centre from Southern Europe). In patients prescribed UCCS (data available in 36 out of 47 patients), 36% (*n* = 13) of centres reported a fasting tolerance <10 h and 64% (*n* = 23) a fasting tolerance >10 h when well. When fasting tolerance was <10 h, the median amount of UCCS given prior to fasting was 1 g/kg (n = 13 patients); the same amount of UCCS was given when fasting tolerance was ≥10 h (*n* = 23 patients). Figure 2 shows the mean dose of UCCS given when fasting is <10 h and >10 h per patient. We did not collect biochemical evidence of fasting tolerance, so it was unclear how respondents determined this or when it was last estimated in individual patients.

### Dietary management when unwell

All centres prescribed an emergency regimen using glucose polymer during illness. The median % glucose concentration from glucose polymer used in emergency feeds during illness was 10% (10-15%) for infants aged 0-12 months; 15% (10-23%) for children aged 1-2y; 20% (10-25%) for children aged 3-10y and 25% (10-30%) in patients over >10y. Only 2 centres used fructose sources such as fruit juice or fruit squash to flavour glucose polymer when glucose polymer was refused. Extra glucose polymer was used in the recovery period in 21 out of 36 centres (58%). Twenty-seven centres (75%) reported that patients accepted oral glucose polymer for their emergency regimen. When it was refused, admission to hospital for IV fluids was advocated.

## Discussion

This is the first paper reporting the dietary management of 126 patients with FBPase deficiency from 36 international centres. With the exception of emergency regimens based on glucose polymer, dietary treatment was not universal with 75% of centres limiting sucrose and fructose and without any relaxation with increasing age. The use of UCCS pre-overnight fast was also not standard practice, although when it was used, a similar amount was prescribed per kg body weight regardless of age and fasting tolerance.

Although it is commonly documented that younger patients have reduced tolerance to fructose, sucrose and sorbitol [4, 15], the necessity for dietary restriction is less clear with age. There is an early case report of an 8 year old who had an acute episode associated with a fructose sweetened cough syrup [19]. Unexpectedly in our survey most patients were prescribed a fructose/sucrose restriction when patients were well. Steinman suggests that patients usually tolerate sweet foods (up to 2 g fructose/kg body weight per day) when given evenly distributed over the day [4], and stringent restriction of fructose intake is unnecessary [4]. Large amounts of fructose especially after a fasting period may lead to metabolic decompensation [20]. It is important to investigate if there are more episodes of metabolic decompensation between patients on or off maintenance dietary restrictions.

It is clear in infancy and young children that fasting times are limited and infants commonly develop clinical symptoms following withdrawal of night feeding: one child died at 3 days of age [1], one developed physical and developmental problems at 3y of age [21] and there are case reports of unexplained hypoglycaemia that stopped at 10y of age [13], but in most cases overall outcome is good. Researchers have previously suggested that fasting tolerance increases with age [2, 15]. Moses et al. [8] suggested that fasting when well is similar to the general population, explained by an increased capacity to store glycogen in the liver, resulting in less dependency of the maintenance of blood glucose on gluconeogenesis. However, symptoms reported in adulthood have been associated with a combination of illness and fasting during Ramadan [22], pregnancy [23], alcohol consumption [24], weight loss, and extensive exercise training (the latter requires careful management) [13].

In 1990, the use of UCCS was reported for the first time by Burlina et al. [25] for 3 cases with FBPase deficiency. They gave 2 g/kg at midnight to prevent hypoglycaemia and since then it has become routine practice in some centres. Although 56% of centres reported its use, a previous UK survey found only 17% of patients were given UCCS [26]. The mean amount of UCCS given in our survey was lower than the dose used by Burlina et al. [25] but there is no efficacy data to support its use or dosage in FBPase deficiency. UCCS has limited benefit when fasting is extended to over 10 h. In our survey, there were no differences in the amounts prescribed for patients with a fasting tolerance over or below 10 h. Glycosade (Vitaflo®) a slow release starch was used by 2 centres, and although it has been shown to be effective in preventing hypoglycemia in patients with glycogen storage disease (GSD) types Ia and Ib [27], its efficacy has not been reported in non-GSD conditions.

There are some limitations in this study. This data was cross-sectional rather than prospective. Data was collected regarding dietary prescription rather than actual patient’s intake and we have no information about patient adherence with daily restrictions. Data on outcome was not collected therefore we were unable to assess if patients on fructose and sucrose restriction had fewer episodes of hypoglycaemia and lactic acidosis which were not associated with illness. No biochemical data was collected on fasting studies to support fasting times and it was unclear how health professionals established fasting times. Most centres only cared for small patient numbers [median *n* = 2 (1-24)].

## Conclusion

This study describes the dietary prescription from responses given to an international questionnaire for one of the largest cohort of patients with FBPase deficiency. This will provide groundwork information to help health professionals when standardising dietary care. Although, the use of an emergency regimen was universal, sucrose and fructose dietary restrictions and the use of UCCS varied widely and particularly in older patients it may be unnecessary. International guidelines on management would optimise dietary treatment preventing any unnecessary over restriction and risk of nutritional deficiencies but also maintaining patient’s safety, which is the primary objective.

## Abbreviations

FBPase: Fructose 1,6 bisphosphatase
GSD: Glycogen storage disease
SSIEM: Society for the Study of Inborn Errors of Metabolism
UCCS: Uncooked cornstarch
